# Experiences and lessons learned from 29 HPV vaccination programs implemented in 19 low and middle-income countries, 2009-2014

**DOI:** 10.1186/s12913-016-1824-5

**Published:** 2016-10-13

**Authors:** Joël Ladner, Marie-Hélène Besson, Etienne Audureau, Mariana Rodrigues, Joseph Saba

**Affiliations:** 1 Epidemiology and Health Promotion Department, Rouen University Hospital, Hôpital Charles Nicolle. 1, rue de Germont, 76 031 Cedex Rouen, France; 2 Axios International, Paris, France; 3 Hôpital Henri Mondor Hospital, Public Health, Assistance Publique Hôpitaux de Paris, Paris Est University, Créteil, France

**Keywords:** Human papillomavirus vaccine, type 16 L1, 18, Vaccination, Preventive health services, Immunization uterine cervical neoplasms, Developing countries, Vaccines, Program evaluation

## Abstract

**Background:**

Cervical cancer is the greatest cause of age-weighted years of life lost in the developing world. Human papillomavirus (HPV) infection is associated with a high proportion of cervical cancers, and HPV vaccination may help to reduce the incidence of cancer. The aim of the study was to identify barriers, obstacles, and strategies and to analyze key concerns and lessons learned with respect to the implementation of HPV vaccination program in low- and middle-income countries.

**Methods:**

The Gardasil Access Program (GAP) is a donation program established to enable organizations and institutions in eligible low-resource countries to gain operational experience designing and implementing HPV vaccination programs. This study used an online survey to capture the experiences and insights of program managers participating in the GAP. Different factors related to HPV vaccination program management were collected. A mixed-method approach enabled the presentation of both quantitative measurements and qualitative insights.

**Results:**

Twenty-nine programs implemented by 23 institutions in 19 low- and middle-income countries were included. Twenty programs managers (97.7 %) reported that their institution implemented sensitization strategies about vaccination prior to the launch of vaccination campaign. The most frequently reported obstacles to HPV vaccination by the program managers were erroneous perceptions of population related to the vaccine’s safety and efficacy. Reaching and maintaining follow-up with target populations were identified as challenges. Insufficient infrastructure and human resources financing and the vaccine delivery method were identified as significant health system barriers. Coupling HPV vaccination with other health interventions for mothers of targeted girls helped to increase vaccination and cervical cancer screening. The majority of program managers reported that their programs had a positive impact on national HPV vaccination policy. The majority of institutions had national and international partners that provided support for human resources, technical assistance, and training and financial support for health professionals.

**Conclusion:**

Local organizations and institutions can implement successful HPV vaccination campaigns. Adequate and adapted planning and resources that support information sharing, sensitization, and mobilization are essential for such success. These results can inform the development of programs and policies related to HPV vaccination in low- and middle-income countries.

## Background

Cervical cancer, the third most common cause of cancer in women around the world, is a significant global health challenge, and is the greatest cause of age-weighted years of life lost in the developing world due to its high incidence [1–3]. Cervical cancer is the second major cause of cancer-related deaths among women in low and middle-income countries (LMICs) [4, 5]. Human Papilloma Virus (HPV) infection is the most common sexually transmitted infection (STI), with a global prevalence of 12 % of women [3, 6, 7]. Two HPV serotypes, HPV- 16 and HPV-18, are found in nearly 70 % of high-grade cervical cancers [2, 8]. Approximately 291 million women worldwide are estimated to have human papillomavirus (HPV) infection of the cervix [6, 7] and the prevalence is higher in women younger than 25 years of age (16.9 %) [2, 9].

Since 2006, two prophylactic HPV vaccines have been available, and each has shown 90 % efficacy in preventing HPV type 16- and 18-associated high-grade cervical lesions [10–12]. While both vaccines are being deployed in developed countries, their use in LMICs has been limited due to cost and a variety of other factors [13, 14]. Recognizing that these factors were impeding the broad use of HPV vaccination in LMICs, Merck & Co., Inc. pledged to donate Gardasil® [Human Papillomavirus Quadrivalent (Types 6, 11, 16 and 18) Vaccine, Recombinant] to eligible LMICs through the Gardasil Access Program (GAP). Axios Healthcare Development is the recipient of this donation and is responsible for managing the GAP. The program was established to enable organizations and institutions in eligible LMICs to gain operational experience designing and implementing HPV vaccination programs, with the goal of supporting the development of successful child and adolescent immunization models [15, 16].

Concerns about the limited use of HPV vaccine in LMICs led to the implementation of a variety of HPV vaccine demonstration projects designed to generate evidence about effective ways to reach young adolescent girls [15, 16]. While these demonstration programs have provided important insights into factors that impact the uptake of HPV vaccine in specific countries and communities, the lessons learned may also be relevant to the implementation of HPV vaccination campaigns in general [17, 18].

Despite the proven safety, efficacy and cost-effectiveness of HPV vaccine, there remain a number of unidentified issues that impede its routine use in LMICs. While a number of HPV vaccination pilot programs have been undertaken in lowest-income countries, published results from these programs do not typically include information related to program management. As more countries consider introducing HPV vaccine, a review of the lessons learned from vaccination programs conducted in LMICs was undertaken in order to identify barriers and difficulties to implementation and sustainability of HPV vaccination programs [17–19]. The experiences of programs participating in the GAP provide additional opportunities to further document and understand the factors related to launching new vaccination interventions in LMICs.

Review of the lessons learned from pilot programs undertaken in LMICs is essential for advancing our knowledge of barriers and solutions to implementing sustainable HPV vaccine programs. The aim of this study was to identify barriers, obstacles, and strategies and to analyze key concerns and lessons learned with respect to the implementation of HPV vaccination in institutions that managed HPV vaccination programs in LMICs through the GAP.

## Methods

Donations through the Gardasil Access Program were available to interested government Ministries of Health (MoH) and national and international non-governmental organizations (NGOs), with the approval of national health authorities. Axios Healthcare Development has been responsible for managing, monitoring, and distributing HPV vaccine for the GAP.

Organizations and institutions participating in the GAP are responsible for covering all other costs associated with their vaccination campaigns, including importation, storage, cold chain management, distribution of vaccine, data collection, and management of the vaccination campaigns. Program managers were independent from Axios and were employees of the organization at which they helped to manage the GAP. The GAP encourages participating organizations to adhere to WHO guidelines for HPV vaccination, which recommends a target age range of 9 or 10 years of age through 13 years of age [20]. The vaccination programs used three models for vaccine delivery: school-based models administered vaccine at local school facilities; health facility-based models administered vaccine at health facilities, hospitals, and mobile clinics; and mixed models used both schools and clinics to deliver vaccine [15, 16]. This study was conducted at the end of the vaccination programs and used a mixed-method approach to provide a descriptive impact evaluation from the perspective of participating GAP institutions. This approach enables the presentation of both quantitative measurements and qualitative insights.

A key method approach of this study was to develop an online survey that could be used to capture the experiences and insights of program managers. The first step in developing this survey was identifying the types of information to be collected. Two health professionals from Axios Healthcare Development (MHB, MR) made in-country site visits to 10 institutions representing 15 programs in 6 countries (Table 1). To ensure representation of countries in Latin America, Asia, and Africa, the programs were randomized and then stratified by continent (Table 1). Structured qualitative interviews were conducted with the program managers, and a variety of relevant topics were explored regarding recruitment, implementation, follow-up, and management of the vaccination programs. The aim was to develop a questionnaire capable of accurately assessing the key challenges related to the implementation of an HPV vaccination program. During the first step of the study, face-to-face interviews were conducted with the program managers in the different sites. The semi-structured interview approach was believed to be the most informative and appropriate for high-level participants, as opposed to a highly- structured questionnaire. This format facilitated the free expression of opinions and ideas among programs managers, and allowed for probing and clarification of responses, and for the identification of new issues and topics relevant in the implementation of an HPV-vaccination program.

**Table 1:** Countries and number of institutions and programs included in the Gardasil Access Program 2009-2014

Country	Online assessment		Site visit assessment
	Number of institutions	Number of programs	Number of institutions
Bhutan	1	1	0
Bolivia	2	4	2
Cambodia	1	2	1
Cameroon	1	1	1
Georgia	1	1	0
Ghana	1	1	0
Guyana	1	1	0
Haiti	1	1	0
Honduras	4	4	4
Kenya	1	1	0
Kiribati	1	1	0
Lesotho	1	2	1
Mali	1	1	0
Moldova	1	1	0
Nepal	1	3	1
Tanzania	1	1	0
Uganda	1	1	0
Uzbekistan	1	1	0
Zambia	1	1	0
Total	23	29	10

The results of these interviews were used to develop the final questionnaire, which was administered online to the program managers of the institutions included in the GAP that completed at least vaccination of the first targeted cohort. The GAP managers at these institutions received a link by email to complete the online standardized questionnaire, which included structured, closed questions, and open-ended questions, in English, Spanish, and French.

Factors related to program management were assessed in the following areas: vaccination sensitization strategies and obstacles reported by parents, identification of obstacles to follow up and strategies to reach girls lost to follow-up, and difficulties related to supply chain management and data management. The number and type of national and international partners that supported the vaccination programs were also collected with respect to technical assistance, logistics, human resources and their training, financial support, and in-kind donations.

Statistical analyses were descriptive, with variables expressed in percentages. Verbatim and qualitative data were collected during the site visits to the 10 institutions, and open-ended questions were included in the final online questionnaire. Qualitative data were systematically analyzed. A directed content analysis approach was used to analyze the data and to identify the key themes. Two authors (JL, MHB) reviewed all qualitative comments extracted from the questionnaires and placed them into broader categories based on content and thematic saturation. Illustrative quotes have been included in this report along with information about the role that each survey participant played in the vaccination program. Key findings and quotes were compiled in a Microsoft Excel table and coded according to topic or theme. Responses were reported if at least two people gave a similar response. Final decisions on comments categories were discussed with a third author (EA).

## Results

A total of 29 programs implemented by 23 institutions in 19 countries are included in this study (Table 1); one program manager (in one institution) has not responded to the survey. In Bolivia, two different institutions implemented four programs; in Honduras, four different institutions managed four programs; in Nepal, one institution managed three programs, and in Cambodia and Lesotho one institution managed two programs in each country (Table 1). Fourteen institutions (60.9 %) were national health institutions of the MoH and nine institutions were international or national NGOs. As specified in the Methods section, 10 institutions in six countries were previously visited as part of the final survey development effort (Table 1). Regarding vaccination delivery models, 18 programs were school-based models, five were health facility-based models, and six were mixed models.

### Sensitization strategies for HPV vaccination

Twenty-two program managers (95.7 %) reported that their institution implemented at least one sensitization strategy about the vaccination program prior to the launch of the campaign; all of the 22 institutions responding implemented sensitization meetings in the communities and organize meetings with the girls (Table 2). The most commonly reported challenges related to vaccination sensitization were misinformation (52.2 %) and a lack of understanding of information (26.1 %) about HPV vaccination.

**Table 2:** Strategies used, obstacles, and difficulties reported by the institutions, Gardasil Access Program 2009-2014 (*N* = 23)

	Number	Percent
Vaccination sensitization strategies		
No sensitization conducted	1	4.3
At least one sensitization conducted	22	95.7
Sensitizations conducted		
Meetings in the communities	22	100
Meetings with girls to be vaccinated	22	100
Media campaigns (radio, TV, newspapers)	17	77.2
Meetings with mothers at time of cervical cancer screening	13	59.1
Obstacles for vaccination reported by the parents		
No obstacle reported	4	17.4
At least one obstacle reported	19	82.6
Obstacles reported		
Vaccination is a way to sterilize the girls	11	57.9
Vaccine is not safe	8	42.1
Vaccination only for girls and not for boys	7	36.8
Vaccination is a family planning medication	5	26.3
Girls aged 9 years are too young to receive vaccination	5	26.3
Vaccination is not effective in preventing HPV infection and cervical cancer	4	21.1
Vaccination gives girls permission to have sex	3	15.8
Strategies to reach girls lost to follow-up		
No strategy implemented	0	0
At least one strategy implemented	23	100
Strategies implemented		
Teachers and school administration contact	17	73.9
Phone calls to girls or family	15	65.2
Community leader contact	13	56.5
Visit to girl at home	8	34.8
Religious leader contact	2	8.7
Girls’ peers	2	8.7
Obstacles for girls’ follow-up		
No challenge reported	3	13.0
At least one obstacle reported	20	87.0
Obstacles reported		
Girls have changed schools or move away	16	80.0
School vacation or examination	11	55.0
Incorrect reporting or lack of details on identity	8	40.0
Limited transportation vehicles available	7	35.0
Limited financial resources available	7	35.0
Limited human resources available	5	25.0
Difficulties of supply chain management		
No difficulty reported	10	43.5
At least one difficulty reported	13	56.5
Difficulties reported		
Vaccines reached expiration date	6	46.2
Difficulties in transporting unused vaccines from sites	5	38.5
Projections of vaccine needed on site did not match number of girls in the field	3	23.1
Insufficient storage capacity at site level	1	7.7
Vaccines damaged during transportation to site	1	7.7
Difficulty of data management		
No difficulty reported	7	30.4
At least one difficulty reported	16	69.6
Difficulties reported		
Reports from vaccination sites were not received in time	14	87.5
Reports from vaccination sites were incomplete or incorrect	7	43.8
The system in place was time-intensive and cumbersome	3	18.8

### Identified obstacles

Nineteen program managers (82.6 %) reported that parents had objections to the HPV vaccine, the most common of which were the misconceptions that HPV vaccination is a method for sterilizing girls (Table 2). Program managers underlined the importance of providing effective messages to parents: *“It is important to have good one-on-one education with parents to overcome misinformation.”* The integration of the HPV vaccine into the Expanded Program of Immunization (EPI) was also found to be an effective message: “*In general parents were open to the HPV vaccine because children received routine immunizations - this helped with acceptance of Gardasil because it was integrated within EPI”.*

The most frequently reported misinformation was related to the reproductive health of girls receiving HPV vaccination, as specified by two program managers: “*Another myth that we had to fight against was that the vaccine was seen as ‘authorization to have sexual relations’”*. Information strategies designed to address these misperceptions remained a crucial point for the programs managers: “*In rural areas we encountered more lack of information, while in urban areas it was more about misinformation*”; “*Understanding myths and knowledge gaps within the communities provided us with the right information to create appropriate messages for education*”; “*The best strategy is to work directly with parents and provide information directly to girls.”*

A total of 21 institutions (91.3 %) required informed consent, including written informed consent (17 institutions, 81.0 %), oral informed consent (2, 9.5 %), and informed consent for the parents to refuse vaccination (2, 9.5 %). Three program managers highlighted sociocultural barriers related to the utilization of informed consent: *“Asking for written consent was taken as a negative sign by parents who became suspicious”; “The best strategy is to work directly with parents, especially for explaining the process and getting consent and making sure they understood the process”; “Providing vaccines free of charge led to parents being more suspicious and less concerned about girls returning for the subsequent doses.”*

### Follow-up strategies

All the institutions were challenged with girls lost to follow-up during the vaccination campaigns. The most frequent obstacles to follow-up were girls changing schools or moving away, and school vacation or examination. One third of the institutions also reported logistical obstacles to follow-up, such as challenges with transportation, and the availability of human and financial resources needed to bring girls to vaccination sites (Table 2). Reaching adolescent girls to deliver vaccines was also reported as a significant challenge, and individual programs used a variety of strategies to address this obstacle. The most frequent strategies were to contact teachers and school administration, and phone calls to girls or her family members. Multiple institutions also used the support of contacts in their communities (Table 2): “*The staff implemented an innovative method of ‘peer tracking’ of girls who needed second and third doses in small communities, some of which did not have cell phone coverage*”; “*Once the team found one girl who needed a second or third dose, they would ask her to help them locate other girls who needed second or third doses*”; “*The process of follow up was costly in terms of time, finances, and human resources*”.

### Management of supply chain and data monitoring systems

Because most of the institutions participating in the GAP have a history of experience delivering other vaccines that are included in the national immunization program or the EPI, management of vaccine supply chain did not pose new challenges, and 43.5 % of program managers reported no difficulties with this aspect of their vaccine campaigns: *“Logistics and cold chain management was apparently not a challenge or issue for any of the projects, apart from some isolated and unexpected events”;* “*Providing supplies and the vaccines in advance (around five days) guarantees the success of the vaccination campaign*”; *“The main challenge appears to be with the storage capacity at some health facilities.”* However, it should be noted that 13 institutions (56.5 %) did report difficulties with HPV vaccine supply chain management, including expiration of vaccine date (46.2 %) and difficulties transporting vaccine to administration sites (38.5 %) (Table 2).

Data management and monitoring and evaluation systems were also investigated. Only one institution aggregated the data rather than collecting individual data for each girl vaccinated. Individual data were recorded in a registry and on individual records in 17 institutions (77.3 %). Of those institutions with individual records, seven (31.8 %) recorded the data in an electronic database. Overall, 82.6 % of institutions managed their programs electronically. Sixteen institutions reported difficulties with the data management of vaccination programs, with late or incomplete vaccination reports listed as two of the most common challenges (Table 2).

### Interventions coupled with HPV vaccination

A total of 13 institutions (56.5 %) coupled HPV vaccination with other health interventions for mothers of targeted girls, including screening of cervical cancer in 12 institutions, tetanus vaccination in one institution, and both cervical cancer screening and tetanus vaccination in one institution. The program managers found this approach to be effective: “*Some of the women participating in these campaigns had their girls vaccinated for HPV, and some of the mothers of the girls who received vaccinations at school ended up receiving screening for cervical cancer”*; *“The mother-daughter approach was effective in reaching out-of-school girls in rural areas.”*

### Lessons learned and impact on public health system

Sixteen institutions (69.6 %) reported that their programs had a positive impact on national HPV vaccination policy: *“The program confirmed that the vaccine was highly acceptable; HPV vaccination was built into the national cervical cancer control strategy”; “The most important result of implementing GAP projects in our country is that we now have the experience of implementing HPV vaccination projects”; “The country benefited from the program: we are now approaching cervical cancer in a comprehensive way, and the GAP led to the establishment of cervical cancer screening centers”; “We had to include Gardasil vaccine issues in the Sexual Reproductive Policy and National Adolescent Health Policy.”*

A positive impact on health policy for cervical cancer screening was also reported: *“After the GAP project, cervical cancer screening projects started in our country”*; *“Not exactly a change in national policy, but the National Commission on Immunization Program has recommended undertaking a study on HPV Vaccination activities. The findings from the GAP program will help to inform decision-making related to the vaccination program”; “The start of HPV projects put the issue of cervical cancer on the table for discussion. Because of these discussions and significant political pressure from different groups, a National Prevention Plan against Cervical Cancer was created in the years following the GAP project. This plan included the use of HPV vaccination as an important strategy for cervical cancer management.”* At the end of the GAP, ten institutions applied for GAVI Vaccine Alliance Program funding: *“The MoH used our experience to apply for GAVI funding.”*

Five institutions (21.7 %) also reported a direct impact on cervical cancer awareness: *“The level of knowledge related to cervical cancer has increased, in particular in those populations where the vaccination took place”; “It has increased the number of women that do their PAP smears.”*

Table 3 presents the number and type of national and international partners that supported the HPV vaccination programs. The most frequent support areas were for human resources (21 institutions; 91.3 %), technical assistance (19; 82.6 %) and training for health professionals and financial supports (18; 78.3 %). Regarding the partners that supported the programs, national Expanded Programs for Immunization were frequently involved in technical assistance, human resources, and training; MoH supported technical assistance and logistics; and Ministries of Education were primarily involved in human resources. International NGOs supported eight institutions (44.4 %).

**Table 3:** Number and type of national and international partners supporting HPV vaccination programs, Gardasil Access Program 2009-2014 (*N* = 23)

Type of supports and partners	Number	Percent
Technical assistance		
Number of partners involved		
0	4	17.4
1	5	21.7
2	7	30.4
≥ 3	7	30.4
Partners involved		
Expanded Program on Immunization	11	57.9
International NGOs	4	21.1
Ministry of Education	2	10.5
Ministry of Health	10	52.6
National NGOs	6	31.6
Other United Nation Agencies	1	5.3
World Health Organisation	3	15.8
Logistics		
Number of partners involved		
0	7	30.4
1	4	17.4
2	5	21.7
≥ 3	7	30.4
Partners involved		
Expanded Program on Immunization	2	12.5
International NGOs	6	37.5
Ministry of Education	1	6.3
Ministry of Health	8	50.0
National NGOs	4	17.4
Other United Nation Agencies	0	0
World Health Organisation	3	81.3
Human resources		
Number of partners involved		
0	2	8.7
1	5	21.7
2	6	26.2
≥ 3	10	43.5
Partners involved		
Expanded Program on Immunization	9	42.9
International NGOs	5	23.8
Ministry of Education	10	47.6
Ministry of Health	2	9.5
National NGOs	9	42.9
Other United Nation Agencies	1	4.8
World Health Organisation	0	0
Training of human resources		
Number of partners involved		
0	5	21.7
1	7	30.4
2	5	21.7
≥ 3	6	26.1
Partners involved		
Expanded Program on Immunization	9	50.0
International NGOs	5	27.8
Ministry of Education	1	5.6
Ministry of Health	6	33.3
National NGOs	4	22.2
Other United Nation Agencies	0	0
World Health Organisation	3	16.7
Financial support		
Number of partners involved		
0	5	21.7
1	8	34.8
2	6	26.1
≥ 3	4	17.4
Partners involved		
Expanded Program on Immunization	2	11.1
International NGOs	8	44.4
Ministry of Education	0	0
Ministry of Health	6	33.3
National NGOs	5	27.8
Other United Nation Agencies	2	11.1
World Health Organisation	1	5.6
In-kind donation		
Number of partners involved		
0	10	43.5
1	8	34.8
2	1	4.3
≥ 3	4	17.4
Partners involved		
Expanded Program on Immunization	2	15.4
International NGOs	5	38.5
Ministry of Education	3	23.1
Ministry of Health	2	15.4
National NGOs	4	30.8
Other United Nation Agencies	0	0
World Health Organisation	1	7.7

## Discussion

This study of 23 key programs managers who were actively involved in the implementation of a variety of HPV programs combines qualitative and quantitative research on the challenges and opportunities associated with implementing the HPV vaccine in LMICs. Our findings document the experiences of various strategies and approaches related to HPV vaccination implementation in LMICs and provide lessons learned and best practices that may be applied to the implementation of other HPV vaccine programs. The most frequently reported obstacles to HPV vaccination were erroneous perceptions that the GAP was using HPV vaccine in the context of a clinical trial, that the HPV vaccine was unsafe, and that the vaccine was being used to sterilize young girls. These misperceptions among parents [20], in concert with rumors and sociocultural barriers, underscore the need for effective and accurate communication about the vaccine’s preventive health benefits, as well as appropriate sensitization and advocacy building among parents and key community stakeholders [21–26]. Education related to HPV as an STI and as a primary cause of cervical cancer is important for mitigating these sociocultural issues [17]. Previously, we demonstrated that the inclusion of key messages regarding the safety and efficacy of the vaccine had a positive impact on vaccination coverage. Key sensitization messages that address safety and efficacy at the launch of a vaccine campaign may help to increase acceptance of HPV vaccination [16].

Reaching and maintaining follow-up with adolescent girls in order to deliver HPV vaccines were challenges for program managers. While vaccination programs have traditionally focused on children less than five years of age, a vaccination such as HPV, which is targeted to adolescents, requires different mobilization and delivery infrastructure. There is currently no experience in mass vaccination in adolescent populations, which may explain the reported challenges. Contacts with schoolteachers and administrators were frequently used as a strategy to reach girls lost to follow-up in the 18 programs that utilized school-based delivery models. School-based projects faced additional challenges when vaccination dates occurred outside of scheduled school days, and it was exponentially more difficult to prevent loss to follow-up in these instances. Despite these challenges, school-based vaccination delivery methods were most effective at reaching girls within the WHO-recommended age range, which is likely due to the fact that girls aged 9-13 years are not usual “clients” of the health care system [27–29]. Of the three types of vaccine delivery models assessed in this study, the school-based model was found to be the strongest positive predictive factor of higher vaccine coverage [14, 16]. One study found that school-based models have also been effective in demonstration programs in Peru, Uganda, Vietnam, and India (vaccine coverage ranged from 82.6 % to 96.1 %) [30]. School absenteeism was one primary difficulty reported by the program managers, suggesting that school-based delivery models are appropriate, but require adequate methods to capture and follow girls who are absent the day of vaccination or who moved away [17, 31].

Community involvement actions appeared to impact the programs with respect to both sensitization strategies before launch of the HPV vaccination campaign as well as reaching girls lost of follow-up once the campaign was underway [16, 24, 26]. The program managers recognized that it was important for key stakeholders to understand, accept, and approve of the benefits associated with HPV vaccination. Understanding which aspects of an HPV vaccine campaign are most influenced by community standards, morals, and expectations may help in developing community engagement actions that impact coverage and adherence to vaccination [16]. Community sensitization about the availability and value of vaccinating school-aged girls against HPV may impact vaccine uptake. A study in Brazil found that the initial method used to notify parents about the vaccine had a significant impact on vaccine indicators [28].

Insufficient infrastructure and human resources financing and the delivery method were identified as significant health system barriers: assistance programs helped to address these factors and support national and international vaccine programs [17–19]. National or international partners supported all the GAP programs included in this study. Given the high proportion of programs in our study sample that used school-based delivery models, Ministries of Education played an important role in supporting the availability of human resources. The most frequent partners were national (MoH and EPI). Eighteen programs were financially supported by and had significant involvement with international NGOs. Our findings suggest that successful implementation of HPV vaccination programs may be feasible in low-resource settings, provided that the health system structure for immunization and national and international financing options are well understood. Delivery strategies built on the strengths of existing national EPI programs that have strong partnerships across multiple healthcare delivery sectors may also enable successful HPV vaccination programs in low-resource settings. Programs such as the GAP were established to donate limited amounts of the vaccine to countries for demonstration programs designed to test HPV vaccine delivery strategies [15, 16]. Such pilot programs suggest that in-country ownership and development capacity may contribute to the long-term success and sustainability of vaccine delivery [16, 17].

The data reported here provide lessons for development of public health programs and policies as countries go forward in national decision-making related to HPV vaccination. These results suggest that local organizations and institutions can implement successful HPV vaccination campaigns. Our results also demonstrate that private initiatives to increase access to novel health interventions can have a significant impact on public health issues and foster diverse partnerships among government agencies, NGOs, and private organizations. The diverse HPV vaccine programs included in this study provide concrete examples of how such programs can be adapted to address local and regional issues and concerns, and provide a useful framework in which countries can consider how best to expand their own HPV vaccination programs based on their individual epidemiological, economic, and health system challenges [22].

The results of HPV vaccine experiences so far in LMICs are encouraging, and the continued sharing of outcomes data, successes, and challenges is critical to the reproducibility of successful HPV vaccine interventions. Considerable progress has been made in several LMICs and it is important to identify and understand those factors that are transferable to other settings. Demonstration and pilot projects in several LMICs have shown that a variety of delivery methods, including the use of schools, special campaigns, health centers or combined strategies, can reach a large proportion of eligible girls, and that advocacy and sensitization are essential elements for success [17, 19, 32].

While our results provide practical information about programs for HPV vaccine introduction in low and middle-resource settings, there are some limitations to be noted. A key limitation of this study is that it represents the experiences and opinions of the program managers of the GAP rather than all the HPV vaccination programs in LMICs. Similarly, there are inherent limitations associated with data that are self-reported rather than obtained through objective assessment or observation. However, the design of our study, which utilized both qualitative and quantitative data collection, and utilized a final study questionnaire that was developed following 10 site visits in six countries and was designed to address potential issues related to self-reported data, should help to limit the bias associated with self-reporting. This study is also limited by the design approach, which measured the impact of the GAP in different areas at the program’s end. This approach does not allow us to conduct analyses on a program-by-program or country-by-country basis, or to compare the quality or type of HPV vaccination programs or other type of vaccine delivery provided before and after implementation of the GAP, which could provide additional validation of the program’s impact on these services. However, with the goal of developing a highly informative online survey, we investigated and developed our questionnaire based on site visits, which provides some level of corroboration between reported and observed results. The results of the survey utilized in this study appear to be robust and do provide important insight into the impact of a donation program on a variety of services and health activities at the local, institutional, and national levels. While the current study does not focus on vaccine delivery models, we previously reported that school and health clinic-based models appeared as predictive factors for vaccination coverage and that HPV vaccine campaigns tailored to meet the needs of communities can be effective [15, 16]. The implementation of the GAP appears to have triggered changes at the national level that may have long-term positive effects.

For LMICs, it is a challenge to accelerate HPV vaccination programs and also to conduct impact analyses around such programs. Considerable progress has been made in several LMICs and it is important to identify and understand lessons that may be transferable to other settings worldwide [17, 18]. School-based programs have been successful [15, 28, 29, 32], but innovative implementation approaches will be necessary to reach broader populations. The suspension of a demonstration HPV-vaccination program, in India following opposition and activism within the community underscores the importance of ensuring that new research approaches engage with the members of the community in which they are undertaken and incorporate societal concerns, public emotions, and local politics. This type of engagement is essential to understand and address the societal challenges of implementing HPV vaccination programs [33]. Developing new alternatives that expand access to HPV and cervical cancer prevention resources will require input from multiple stakeholders, including governments, NGOs, the scientific community, drugs companies, and communities [33]. More research is needed on several practical issues, such as how to increase access to HPV vaccines and to develop the implementation of HPV vaccination programs in LMICs. This study could provide insight into strategies that could be used to determine best practices related to sensitization strategies, stigma avoidance, engaging with the community, and follow-up with young girls during the vaccination campaign in particular socio-cultural contexts.

## Conclusion

Routine vaccination in adolescents remains challenging. The data reported here from programs focused on vaccinating adolescent girls provides lessons that may be applied to the development of public health programs and policies as countries go forward in national decision-making for HPV vaccination. Our findings in a large sample of HPV vaccination programs demonstrate that continued sharing of evidence, successes, and challenges is critical to the successful scale-up of HPV vaccine interventions worldwide. These findings underscore the need for adequate and adapted planning and resources that support information sharing, sensitization, and mobilization. The GAP provides a model for how private initiatives can have a significant impact on public health issues.

The diverse HPV vaccine programs included in this study provide concrete examples of how such programs can be adapted to address local and regional issues and concerns, and provide a useful framework in which countries can consider how to best expand their own HPV vaccination programs based on their individual epidemiological, economic, and health system challenges.

## Abbreviations

EPI: Expanded programme on immunization
GAP: Gardasil access program
HPV: Human papilloma virus
LMICs: Low and middle-income countries
MoH: Ministry of Health
NGO: Non-Governmental Organization
STI: Sexually Transmitted Infections
UN: United Nations
WHO: World Health Organisation
